# Supplementary material for: Biomimetic Calcium Phosphate Coatings for Bioactivation of Titanium Implant Surfaces: Methodological Approach and In Vitro Evaluation of Biocompatibility
Source: Materials (Basel). 2021 Jun 24;14(13):3516. doi: 10.3390/ma14133516 (PMC8269522; doi:10.3390/ma14133516)
Supplement: Supplementary file 1 [file materials-14-03516-s001.zip › materials-1255683-supplementary.pdf]

Supplementary

Biomimetic Calcium Phosphate Coatings for Bioactivation of Titanium Implant Surfaces: Methodological Approach and In Vitro Evaluation of Biocompatibility

Thomas Kreller [1], Franziska Sahm [2], Rainer Bader [2], Aldo R. Boccaccini [1], Anika Jonitz-Heincke [2] and Rainer Detsch [1,*]

Table S1. Order and Amount of reagents for 1 l of BCPx1, BCPx1.5, and BCPx2.

Order	Material	Amount BCPx1	Amount BCPx1.5	Amount BCPx2
1	NaCl	8.035 g	12.053 g	16.070 g
2	$NaHCO_3$	0.355 g	0.533 g	0.710 g
3	KCl	0.225 g	0.338 g	0.450 g
4	K_2HPO_4 $3H_2O$	0.231 g	0.347 g	0.462 g
5	$MgCl_2$ $6H_2O$	0.311 g	0.467 g	0.622 g
6	HCl	39 mL	53 mL	66 mL
7	$CaCl_2$ $2H_2O$	0.292 g	0.438 g	0.584 g
8	Na_2SO_4	0.072 g	0.108 g	0.144 g
9	Tris	6.118 g	9.177 g	12.236 g
10	HCl	0–5 mL	0–5 mL	0–5 mL

Table S2. Equations.

$$\text{Crystallinity [\%]} = \frac{A_C}{A_{HA}} \text{ x } 100\ \%. \quad (1)$$

$$\textit{Crystallite size [nm]} = \frac{Kx\lambda}{\beta \cos\theta} \quad (2)$$

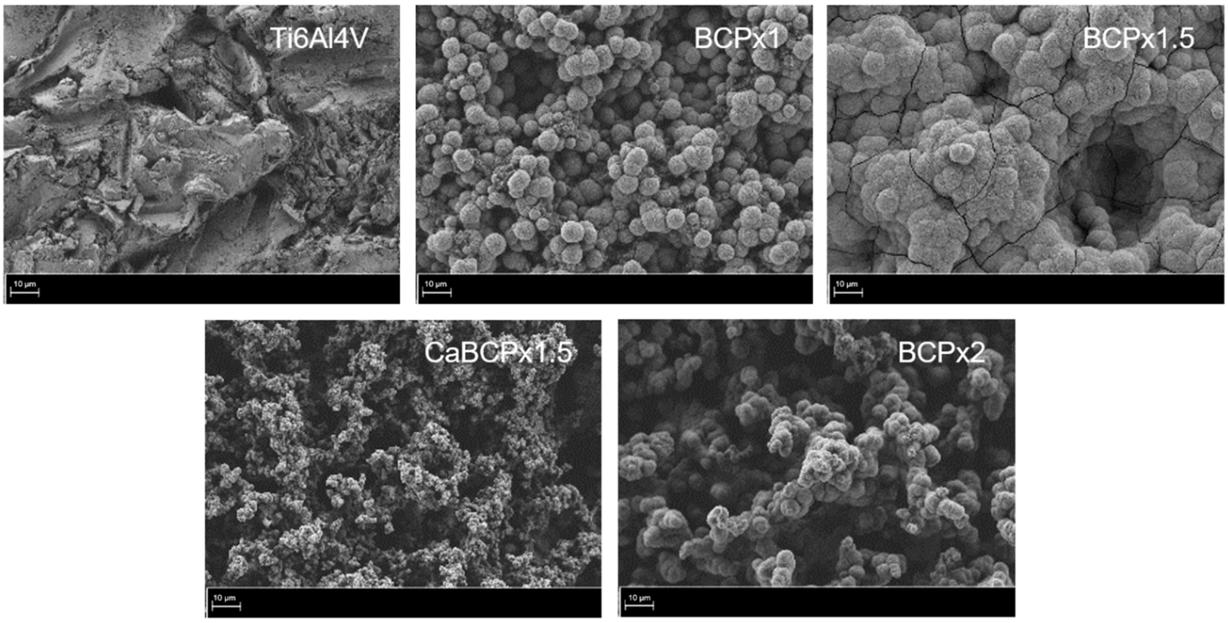

Figure S1. SEM analysis of biomimetic coatings precipitated on variable chemically pre-treated Ti6Al4V substrates after exposure to BCPx1, BCPx1.5, and BCPx2 for 14 days. Overview Images, Scale: 10 µm.

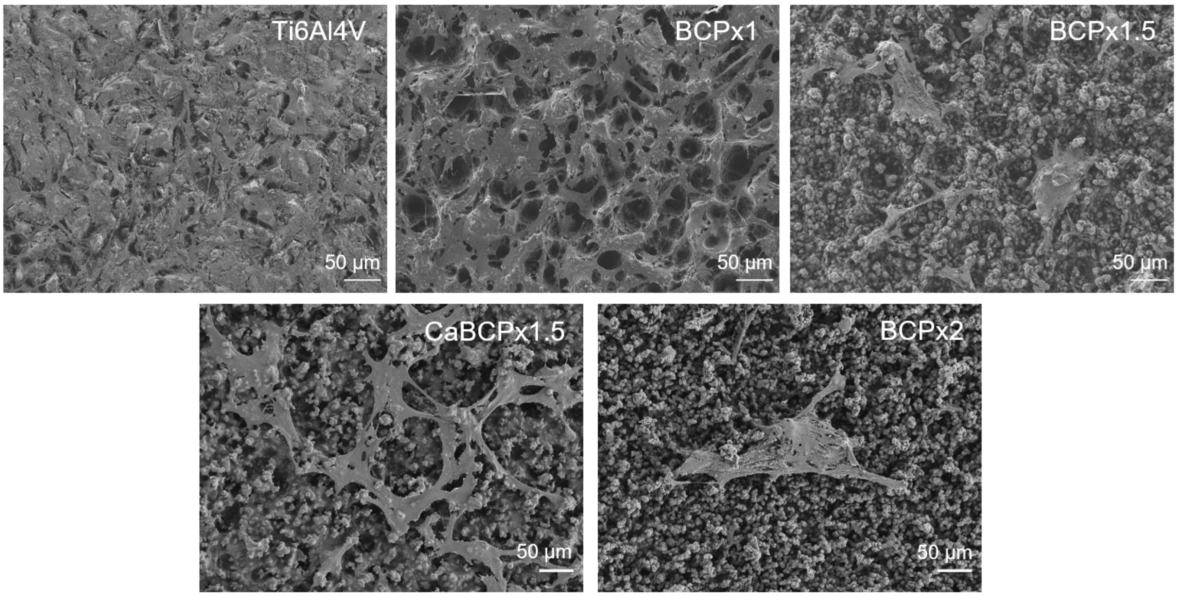

Figure S2. Cell morphology of human osteoblasts growing on coatings precipitated on chemically pre-treated Ti6Al4V substrates after exposure to BCPx1, BCPx1.5 with and without CaCl2 pre-treatment, and BCPx2 for 14 days. Images were taken 48 h after cell seeding with an SEM, scale: 50 µm.
